# Implementing referral to an electronic alcohol brief advice website in primary healthcare: results from the ODHIN implementation trial

**DOI:** 10.1136/bmjopen-2015-010271

**Published:** 2016-06-16

**Authors:** Preben Bendtsen, Ulrika Müssener, Nadine Karlsson, Hugo López-Pelayo, Jorge Palacio-Vieira, Joan Colom, Antoni Gual, Jillian Reynolds, Paul Wallace, Lidia Segura, Peter Anderson

**Affiliations:** 1 Department of Medical Specialist and Department of Medical and Health Sciences, Linköping University, Motala, Sweden; 2 Department of Medical and Health Sciences, Linköping University, Linköping, Sweden; 3 Grup Addiccions Clínic (GRA-GRE), Hospital Clínic de Barcelona, IDIBAPS, RTA, University of Barcelona, Barcelona, Spain; 4 Program on Substance Abuse, Public Health Agency, Government of Catalonia, Barcelona, Spain; 5 Department of Primary Care and Population Health, University College London, London, UK; 6 Institute of Health and Society, Newcastle University, Newcastle upon Tyne, UK; 7 Faculty of Health, Medicine and Life Sciences, Maastricht University, Maastricht, The Netherlands

**Keywords:** Alcohol screening, brief intervention, referral to electronic brief advice, fidelity to intervention

## Abstract

**Objectives:**

The objective of the present study was to explore whether the possibility of offering facilitated access to an alcohol electronic brief intervention (eBI) instead of delivering brief face-to-face advice increased the proportion of consulting adults who were screened and given brief advice.

**Design:**

The study was a 12-week implementation study. Sixty primary healthcare units (PHCUs) in 5 jurisdictions (Catalonia, England, the Netherlands, Poland and Sweden) were asked to screen adults who attended the PHCU for risky drinking.

**Setting:**

A total of 120 primary healthcare centres from 5 jurisdictions in Europe.

**Participants:**

746 individual providers (general practitioners, nurses or other professionals) participated in the study.

**Primary outcome:**

Change in the proportion of patients screened and referred to eBI comparing a baseline 4-week preimplementation period with a 12-week implementation period.

**Results:**

The possibility of referring patients to the eBI was not found to be associated with any increase in the proportion of patients screened. However, it was associated with an increase in the proportion of screen-positive patients receiving brief advice from 70% to 80% for the screen-positive sample as a whole (p<0.05), mainly driven by a significant increase in brief intervention rates in England from 87% to 96% (p<0.01). The study indicated that staff displayed a low level of engagement in this new technology. Staff continued to offer face-to-face advice to a larger proportion of patients (54%) than referral to eBI (38%). In addition, low engagement was seen among the referred patients; on average, 18% of the patients logged on to the website with a mean log-on rate across the different countries between 0.58% and 36.95%.

**Conclusions:**

Referral to eBI takes nearly as much time as brief oral advice and might require more introduction and training before staff are comfortable with referring to eBI.

**Trial registration number:**

NCT01501552; Post-results.

**Strengths and limitations of this study**

- There is a lack of studies on implementing referral to an alcohol electronic brief intervention (eBI) by healthcare staff in primary healthcare as reported in the present study.
- The strength of this study is the participation from five jurisdictions, enabling us to study the variability of referrals to eBI.
- In addition, the high number of participating providers and primary healthcare units (PHCUs) is seen as a strength.
- Limitations include the failure of some jurisdictions to implement referral to the eBI as intended, as well as the lack of access and trust in internet-based health promotion among patients (that might be due to the age of the population screened in some jurisdictions).

## Background

Alcohol continues to be a leading cause of disease globally.^1^ Despite evidence on the efficacy and cost efficacy of screening and brief advice to risky drinkers in primary healthcare, these interventions are rarely implemented in routine practice, resulting in identification of <10% of the population at risk and <5% of those who are screened receiving brief advice.^2–4^ Although delivery of a brief alcohol intervention might only take 10–15 min, this is too time-consuming for most consultations and has been put forward by healthcare professionals as one of the key factors hindering more widespread implementation of brief alcohol interventions.^2^ ^5–7^

As access to the internet has increased, electronic brief advice websites (electronic brief intervention (eBI)) for risky drinkers have been developed and made available online. Research indicates that they can have beneficial effects equivalent to face-to-face interventions depending on the content of the eBI and the target group.^8–11^ Such interventions might reduce the workload of healthcare professionals after identification of patients with risky drinking and could be effective if the patients respond positively when advised by their healthcare professionals to use the online intervention.

Optimising the use of electronic health behavioural interventions in the population at large is a major challenge.^12^ In a review on what enhances exposure to online interventions using various methods of promotion, it was suggested that referral or promotion by a health professional might be an effective means of increasing the use of online interventions.^13^ There is an increasing amount of literature on the feasibility of guided or facilitated access to online interventions for anxiety and depression; there are fewer studies on facilitated access to online alcohol interventions.^14^

One of the few published studies involving the offer of an eBI in a primary care setting was undertaken in the UK where staff at 18 primary healthcare centres agreed to refer patients with risky drinking to an electronic alcohol intervention.^15^ Referral to the intervention after identification of risky drinking behaviour was done in two stages. Over a period of 12 months, these 18 practices managed to refer a total of just 31 patients, of whom only 19 actually attended a first face-to-face appointment (first step) and 6 eventually logged on to the intervention website (step 2). The two-stage referral process in this study may have played a significant role in the low rates of access to the eBI. However, the study highlights the various challenges in initiating discussions about alcohol in practice. This was partly explained by the reluctance of the staff to screen for risky drinking and difficulty in remembering the possibility of referring to an online intervention.

Research on referral of patients by healthcare staff to internet applications promoting healthy lifestyle such as low-risk drinking is still in its infancy; only a few studies have been conducted in the UK and Sweden, and a few more studies are underway.^16^

The Optimizing Delivery of Health Care Interventions (ODHIN) trial was designed to evaluate the effect of three implementation strategies (alone or in combination) on implementation of alcohol screening and brief intervention in primary healthcare: (1) financial reimbursement (FR), (2) training and support (TS) and (3) facilitated access to an eBI as an alternative to face-to-face intervention.^5^ The trial was an eight-arm factor cluster randomised controlled trial (RCT) in which facilitated access to an eBI was included in four of the eight arms, with or without one or more of the other interventions. The trial was undertaken in 120 primary healthcare units (PHCUs), distributed equally across the five participating jurisdictions. This article reports the findings from the study with specific reference to referral to eBI.

The objective of the present study was to explore whether the possibility of offering facilitated access to an eBI instead of delivering oral brief advice in the ODHIN study increased the proportion of consulting adults who were screened and the proportion given brief advice using data from the ODHIN study. The study also examines differences in the levels of implementation among the various participating jurisdictions in the trial and adherence to referral to eBI among the patients.

## Participants and methods

This study is a subanalysis of the data obtained during the ODHIN RCT.^5^ ^17^ We used data obtained during the trial relating to the offer of facilitated access to an eBI as an alternative to providing a face-to-face brief intervention to risky drinkers.

The results of the 12-week implementation of the ODHIN RCT will be described in a forthcoming article (P Anderson *et al*. Submitted to *Addiction*). A total of 120 PHCUs in Catalonia, England, the Netherlands, Poland and Sweden were randomly allocated to one of the eight groups using computerised randomisation, stratified by jurisdiction. In total, 746 individual providers (general practitioners, nurses or other professionals) agreed to participate in the trial and gave written informed consent after a 30–45 min introductory meeting explaining the evidence base on alcohol screening and brief interventions.

Of the 120 centres, 15 were allocated to the eBI strategy alone and 45 to combined intervention strategies (TS, FR and TS plus FR (TS+FR)). After formal agreement with the PHCU to take part in the trial, baseline measurements took place over a 4-week period. After a gap of 2–6 weeks, the 12-week implementation period began; the start date for each jurisdiction was between November 2012 and May 2013. All seven intervention groups received the same basic input as the controls together with additional components. Providers in PHCUs allocated to referral to eBI (alone or in combination) had a mean age of 47.1 years (SD=9.4; range 24–67 years) and 76% were women. The mean age of the providers was lowest for the Netherlands (44.1 years) and highest for Poland (48.9 years). The proportion of women varied from 68% in the Netherlands to 90% in Sweden.

Staff in each PHCU were asked to screen adult patients (≥18 years of age who attended the PHCU for any reason) for risky drinking, using a paper version of the Alcohol Use Disorders Identification Test—Consumption (AUDIT-C)^18^ except in Catalonia, where a computerised version was used. On the tally sheet (or in Catalonia, the electronic record), any brief intervention activity was to be recorded.

Screen positives were defined in Catalonia and England as men and women who scored ≥5 on AUDIT-C, and in Poland, the Netherlands and Sweden as men who scored ≥5 and women who scored ≥4 on AUDIT-C. The providers in all eight arms were asked to deliver brief alcohol advice to screen positives, with the length and format of the advice based on country-specific guidelines or existing routines. In addition, the eBI arms were given the option of referring to an eBI website. No demographic data concerning the patients were collected.

Providers in PHCUs allocated to referral to eBI (alone or in combination) were offered the opportunity to refer patients to an online brief alcohol intervention as an alternative to a face-to-face brief intervention, but were advised that they could continue to offer face-to-face intervention if they so wished to do so. Providers were advised that referral should consist of taking a few minutes to encourage screen-positive patients to log on to the designated eBI package. All providers in this arm of the trial were trained in how to offer the eBI to the patients using a designated script and were asked to spend time familiarising themselves with the website before referring patients to the intervention. Unique log-on codes were used to trace whether they actually did so. Patients referred to the eBI were handed a leaflet containing a unique log-on code for an approved eBI online intervention. The eBI website used in each jurisdiction was required to meet the following criteria: (1) customised as an ODHIN website, (2) log-on facility to allow monitoring of patient log on, (3) suitable brief screening tool with the ability to calculate a score and give feedback (ie, intervention), (4) appropriate information on sensible drinking guidelines, (5) information on the impact of alcohol on health and well-being and (6) a drink diary facility. In each country, the eBI package was selected from existing programmes with the exception of Poland where the WHO eBI programme was used.

### Measures

The tally sheet with the AUDIT-C score and registration of brief advice activity (or for Catalonia, the electronic record) was used to calculate the proportion of patients screened, the proportion of screen-positive patients who were offered brief advice in any of the following formats: oral advice, given an informative leaflet about sensible drinking, referral for oral advice by other staff or referral to eBI.

The staff were asked to indicate on the tally sheet which forms of brief advice activity were offered to the patients. In jurisdictions using paper tally sheets, it was possible to tick (and therefore deliver) several forms of brief advice to one patient in a single consultation, whereas in Catalonia, just one option was recorded on the electronic record. Staff were also asked to note on the tally sheet if the patient did not want to be referred to eBI or had no computer, and to take note of any other reason for not offering advice during the consultation, including lack of time. On the paper version, it was possible to tick more than one activity, but for Catalonia, only one option could be selected.

The proportion of patients screened was calculated as the number of patients screened divided by the number of patients eligible for screening per participating provider times 100.

The proportion of screen-positive patients given brief advice was calculated as the number of screen-positive patients who received oral brief advice and/or were given a leaflet, were referred to another provider in or outside the practice or were referred to eBI divided by the total number of screen-positive patients per participating provider times 100.

The proportion of patients logging on to the eBI was calculated as the number of patients who logged on to the intervention per PHCU divided by the number of eBI referral cards handed out (calculated from the tally sheets of each PHCU) times 100. The number of patients logging on to the eBI was retrieved from the eBI system in each jurisdiction. Each patient could be traced using the log-on number unique to an individual provider.

After the implementation period, staff were asked to complete a questionnaire as part of a monitoring process of attitudes to dealing with heavy drinkers that was established throughout the ODHIN trial. This questionnaire also enquired about the average time spent delivering screening and oral advice as well as screening and referral to eBI.

### Statistical analysis

For descriptive purposes, the proportion for categorical variables and the mean values for quantitative variables were calculated. Differences in the proportion of providers familiarising themselves with the content of the eBI website were compared between active and non-active providers using the χ^2^ test.

The main ODHIN trial had a factorial design (P Anderson *et al*. Submitted to *Addiction*), in which (−1,1) coding was used, resulting in the outcome regression coefficients having half the effects. The eBI factor was coded as follows: eBI=−1 for the control, FR, TS and TS+FR arms and +1 for the eBI, eBI+TS, eBI+FR and eBI+TS+FR arms.

Analyses were performed with IBM SPSS V.22, using the MIXED procedure with a random intercept and fixed variables that included the factor and baseline measurements. Owing to the hierarchical structure of the data (provider within PHCU within jurisdiction), models were analysed with PHCU nested within country as random-effect variables. Evidence for interactions between TS, FR and eBI was investigated. There was an interaction between FR and eBI for screening rates, and the interaction term FR×eBI was entered in the models. There was an interaction between TS, FR and eBI for brief advice rates, and the interaction term TS×FR×eBI was entered in the models. The outcome rates were estimated marginal means per provider with 95% CIs, accounting for provider within PHCU within jurisdiction. When examining the impact of the factors on the 12-week screening and brief advice rates, examination of residuals found them to be not symmetrically distributed around 0, so log-transformed data, which provided a better fit, were used. Before logging, rates with a value of 0 were assigned a value of 0.001. Differences in the coefficients with and without the factor were tested by t-tests in the MIXED procedure.

### Ethical approval

Ethical approval for the study was obtained within each jurisdiction from the relevant approval bodies (P Anderson *et al*. Submitted to *Addiction*).

## Results

In total, 350 providers from 60 PHCUs were allocated to one of the four arms that included eBI referral. Of these, 178 (51%) providers from 56 different PHCUs participated actively in referral of patients to the eBI by handing out at least one eBI referral card to the patients. The remaining 172 (49%) providers from 41 different PHCUs did not hand out any eBI referral cards.

Of the 350 providers, 252 (72%) never familiarised themselves with the content on the eBI website. Of the 178 active providers referring patients to the eBI, 71 (40%) familiarised themselves with the eBI website in contrast with the 172 non-active providers, of whom only 27 (16%) logged on to the website (χ^2^ 25.39, df 1, p=0.0001).

During the 12-week implementation period, a total of 3405 (35.4%) of the 9619 patients screened were found to have a positive AUDIT-C score and of these, 1286 (38%) from 56 of the 60 PHCUs were referred to eBI.

Calculations for the proportion of referred patients logging on to the eBI were obtained from 54 of the 60 PHCUs, excluding 4 PHCUs that did not refer any patient to eBI and 2 PHCUs for whom no log-on rate was reported (missing value). A total of 17 of the 60 PHCUs had a log-on rate of 0 despite having referred patients to the eBI; 9 PHCUs in Catalonia, 5 in the Netherlands and 3 in Poland.

The number of providers, referrals to eBI and mean log-on rates are presented in table 1. The mean log-on rate was 18.40% based on values from 54 of the 60 PHCUs.

**Table 1:** Number of referrals to eBI and log-on rates per jurisdiction in 60 PHCUs randomised to the eBI arms of the ODHIN trial

Jurisdiction	Providers, n	Active providers, n (%)*	Referrals to eBI, n†	Mean log-on rate (%)
Catalonia	107	34 (32)	100	0.58
England	52	39 (75)	258	28.81
The Netherlands	72	28 (39)	58	17.32
Poland	34	33 (97)	793	10.58
Sweden	85	44 (52)	198	36.95
Total	350	178 (51)	1407	18.40

*Active providers defined as those who had handed out at least one eBI referral card during the 12-week implementation period.

†Number of patients referred to eBI.

eBI, electronic brief intervention; ODHIN, Optimizing Delivery of Health Care Interventions; PHCUs, primary healthcare units.

### Change in screening and brief advice rate between baseline and after implementation

One of the aims of the study was to explore whether the possibility of referring to the eBI would increase the proportion of patients screened and receiving brief advice (among screen positives). In general, little evidence of this was found (table 2). The eBI was not associated with any increase in the proportion of patients screened but was associated with an increase in the proportion of patients receiving brief advice in the sample as a whole (p<0.05) and in England in particular (p<0.01).

**Table 2:** Mean proportion of patients screened and proportion given brief advice (95% CI) per provider at baseline and after the implementation period without and with each eBI factor, including all 60 PHCUs randomised to the eBI arms

		Proportion screened, % (95% CI)†		Proportion given brief advice, % (95% CI)‡	
		Baseline	Implementation	Baseline	Implementation
Catalonia	Without eBI option	7.3 (4.4 to 10.2)	8.4 (6.4 to 10.4)	52.5 (34.9 to 70.0)	69.0 (58.9 to 79.1)
	With eBI option	8.6 (5.6 to 11.6)	8.5 (6.4 to 10.7)	47.4 (27.4 to 67.4)	67.7 (56.5 to 78.8)
England	Without eBI option	5.4 (3.3 to 7.5)	8.0 (4.8 to 11.1)	83.2 (71.4 to 94.9)	82.3 (72.4 to 92.2)
	With eBI option	4.6 (2.4 to 6.8)	3.7 (0.5 to 6.9)	86.8 (75.3 to 98.2)	96.0 (86.0 to –100.0)**
The Netherlands	Without eBI option	11.5 (6.9 to 16.1)	8.7 (4.6 to 12.8)	80.6 (72.3 to 89.0)	74.3 (65.3 to 83.2)
	With eBI option	8.8 (4.0 to 13.6)	5.8 (1.5 to 10.1)	66.3 (57.0 to 75.5)	77.6 (68.3 to 87.0)
Poland	Without eBI option	3.4 (0.5 to 6.2)	24.4 (14.3 to 34.4)	94.7 (87.1 to 100.0)	91.1 (85.2 to 97.0)
	With eBI option	1.3 (0 to 4.1)	13.0 (3.0 to 22.9)	96.6 (87.9 to 100.0)	91.6 (86.2 to 97.1)
Sweden	Without eBI option	13.6 (0 to 59.8)	11.9 (3.2 to 20.6)	75.1 (60.1 to 90.2)	73.4 (61.4 to 85.4)
	With eBI option	48.2 (2.7 to 93.6)	16.7 (8.1 to 25.2)	67.9 (53.8 to 82.0)	78.5 (66.6 to 90.3)
Total	Without eBI option	8.6 (0 to 18.9)	10.8 (8.3 to 13.4)	73.9 (67.0 to 80.8)	76.4 (71.5 to 81.2)
	With eBI option	16.2 (5.6 to 26.8)	9.6 (7.0 to 12.3)	70.4 (63.2 to 77.5)	80.7 (75.7 to 85.7)*

See the Statistical analysis section for an explanation of the statistical tests. The tests examine differences in implementation rates in the presence of a factor compared with the absence of a factor, controlling for baseline rates, accounting for the multilevel nature of the data (providers nested within PHCUs nested within countries). *p<0.05; **p<0.01.

†Proportion screened was calculated as the number of patients screened divided by the number of patients eligible for screening per participating provider times 100.

‡Proportion given brief advice was calculated as the number of screen-positive patients who received oral brief advice, and/or were given a leaflet, were referred to another provider within or outside the practice or referred to eBI, divided by the total number of screen-positive patients per participating provider times 100.

eBI, electronic brief intervention; PHCUs, primary healthcare units.

Providers who had familiarised themselves with the content on the eBI website had a slightly higher proportion of screening during the 12-week implementation period, controlling for baseline proportions (11.5%; 95% CI 8.0% to 15.4%), than providers who had not (8.8%; 95% CI 6.1% to 11.4%), but this difference was not significant (t=1.4, NS).

### Use of referral to the eBI in relation other BI activities

Although the providers in the eBI arm were encouraged to refer patients to the eBI, they were still able to offer face-to-face advice, hand out a leaflet about sensible drinking and/or refer patients to other staff at the PHCU or outside the PHCU, who would then deliver oral advice. The distribution of the type of advice offered in the eBI arms among AUDIT-positive patients is presented in table 3, including the number of patients who refused referral to the eBI or did not have a computer or were referred to other staff/did not want advice. Several brief advice formats could be used and delivered in combination within the same consultation in countries using paper tally sheets; in Catalonia, providers could only record one brief advice option.

**Table 3:** Number of patients with a positive AUDIT screening receiving each type of advice* per participating jurisdiction in the 60 PHCUs in the eBI arms

Jurisdiction	Number of patients with a positive screening	Patients receiving oral advice, n (%)	Patients referred to eBI, n (%)	Patients handed a leaflet, n (%)	Patients not accepting eBI/no computer, n (%)	Patients referred to other staff (within or outside the practice), n (%)	Patients referred to another kind of treatment or consultation, n (%)	Patients who did not get advice due to lack of time, n (%)
Catalonia	492	138 (28.1)	74 (15.0)	110 (22.4)	25 (5.1)	9 (1.8)	1 (0.2)	2 (0.4)
England	817	601 (73.4)	245 (30.0)	153 (18.7)	97 (11.9)	43 (5.3)	39 (4.8)	20 (2.5)
The Netherlands	546	323 (59.2)	55 (10.1)	19 (3.5)	42 (7.7)	28 (5.1)	9 (1.7)	57 (10.4)
Poland	964	462 (47.9)	754 (78.2)	98 (10.2)	69 (7.2)	13 (1.4)	3 (0.3)	6 (0.6)
Sweden	586	313 (53.4)	158 (27.0)	86 (14.7)	58 (9.9)	17 (2.9)	39 (6.7)	29 (5.0)
Total	3405	1837 (54.0)	1286 (37.8)	466 (13.7)	291 (8.6)	110 (3.2)	91 (2.7)	114 (3.3)

*More than one option could be selected except in Catalonia where it was possible to tick only one box.

AUDIT, Alcohol Use Disorders Identification Test; eBI, electronic brief intervention; PHCUs, primary healthcare units.

A little more than half of the 3405 cases with a positive AUDIT score received face-to-face advice (n=1837), whereas about one-third (1286) were referred to the eBI.

Separate analysis showed that about half of the patients (608) referred to the eBI received this as the only intervention activity. For each jurisdiction, the proportion of referrals to the eBI as the only intervention was as follows: Catalonia 15.0%, England 7.1%, the Netherlands 3.3%, Poland 41.1% and Sweden 10.6%. However, for Catalonia, only one option could be selected on electronic tally sheet.

Furthermore, among the 1837 patients receiving face-to-face advice, 616 (34.6%) were also referred to eBI. The log-on rate was somewhat higher among these patients (22.0%; CI 18.5% to 25.5%) compared with those only being referred to the eBI (16.9%; CI 13.2% to 20.6%). A total of 8.6% of the patients refused referral to the eBI or rejected this option because of lack of access to a computer. Only a small minority of the patients were referred to other staff within or outside the practice, meaning that, in most cases, the staff who screened the patients also delivered the advice. Only 114 (3.3%) of the patients who screened positive did not get any advice because of lack of time.

The distribution of the various forms of interventions offered is presented by jurisdiction in table 3. Providers in Poland had the highest uptake of eBI referrals with nearly 80% of their patients with a positive AUDIT screen being referred to eBI. The lowest proportion of referrals to eBI was seen in the Netherlands where only 10% were referred to eBI. In all jurisdictions except Poland, the most frequently used intervention was face-to-face advice, which was given on average to 54% of screen-positive patients, ranging from 28% in Catalonia to 73% in England.

### Time spent delivering face-to-face advice and eBI

One of the reasons for including eBI as an arm in the trial was an expectation that referral to an eBI advice system would take less consultation time than delivering brief advice face to face. However, this was not found to be the case. Although the mean self-reported time per provider for referring a screen-positive patient to an eBI programme (5.5 min, 95% CI 4.4 to 6.5) for those providers in the eBI arms was less than the mean time (7.0 min, 95% CI 5.8 to 8.1) for delivering face-to-face brief advice for those providers not in an eBI arm, this difference, accounting for the multilevel nature of the data, was not significant (t=1.90, p=0.06). When examining the time differences by country, time spent on referral to an eBI advice system was significantly less than time spent delivering face-to-face brief advice in Poland (5.0 min vs 7.8 min, p<0.05) and Sweden (4.5 min vs 8.6 min, p<0.05). However, these time benefits were not associated with improvements in screening or brief advice rates in either Poland or Sweden (table 2).

## Discussion

### Main findings

The study found little evidence to support the main hypothesis that the possibility of referring to an eBI would increase the proportion of patients screened and receiving brief advice. The eBI was not associated with any increase in the proportion of patients screened. However, it was associated with an increase in the proportion of screen-positive patients receiving brief advice from 70% to 80% of the screen-positive sample as a whole (p<0.05), mainly driven by a significant increase in England from 87% to 96% (p<0.01).

Furthermore, the findings in the study indicate that staff displayed a low level of engagement and perhaps mistrust in this new technology. Only 28% of all the participants in the eBI arms of the trial familiarised themselves with the eBI; this proportion increased to 40% among those who referred at least one patient (51% of the providers), suggesting that those who were familiar with the eBI package were more likely to recommend its use. Staff continued to offer face-to-face advice to a larger proportion of the patients (54%) than referring to the eBI (38%).

A wide variability in engagement (on average, 18% of referred patients logged on to the website) was seen among patients both between and within jurisdictions.

Our findings are in line with previous research that has repeatedly shown the challenges of competing tasks (ie, other important things that have to be done as part of the daily routine) facing staff in primary healthcare are often a hindrance when implementing new methods, not least implementing new methods for working with risky drinkers.^19^ ^20^ This was also suggested as the main reason for the low implementation rate in a previous qualitative study on referral to an online intervention in primary care in the UK.^15^ Also, the new technology might not fit with the professional's views on how to interact with patients.^21^ Referral to the eBI thus challenges roles and responsibility with regard to the patients' health and uncertainty about the potential benefits to the patients. Also, as shown in this study, referral to eBI takes just as much time as delivering a face-to-face intervention.^22^ We did not explore potential reasons for non-compliance with the study protocol, but certainly this needs to be explored in future research.^22^

### Adherence to the intervention among referred patients

Engagement of the referred patients varied to a high degree both within and across jurisdictions. In two jurisdictions, Catalonia and the Netherlands, a high number of PHCUs had a log-on rate of 0, that is, patients did not log on, despite having being referred to the eBI. However, in Poland, and in particular in England and Sweden, eBI referrals seem to have been implemented without any major problems (table 1). Lack of adherence to or attrition from online health interventions is a well-known problem.^15^

Facilitated access or referral has been shown to reduce attrition in psychological online interventions targeting anxiety and depression.^23–25^ In our study, the mean proportion of patients adhering to the referral was low, on average 18.4%, but in one-third of the PHCUs, no patients logged on to the website. However, two jurisdictions showed more positive results with adherence of 37% in Sweden and 29% in England. In one of the few earlier studies on primary healthcare referral to eBI in the UK, adherence was 32% based on only 19 referrals.^15^ Despite the lack of results from some PHCUs in our study, the results indicate that it is feasible to refer patients to an eBI and get a reasonable proportion of them to log on, although the results also point to implementation issues that need to be considered and explored in more depth in future studies. We did not specify a mandatory revisit to the provider, which might have increased the adherence rate somewhat.

Our findings concerning the engagement of the patients are in line with a recent review in which it was concluded that the few projects published so far on referral to eBI have not been able to show a satisfactory level of engagement and sustainability. Referral to eBI seems only to succeed if the healthcare provider offers personal engagement in promoting the referral, ensuring that the patients log on and adhere to the online intervention.^16^

### Use of referral to eBI in relation to other BI activities

Staff in this study were given the option of referring patients to the eBI, but they could also offer face-to-face advice, hand out a leaflet or refer patients to another professional within or outside the PHCU, except for Catalonia where only one option could be recorded in the electronic record. Only half of the patients referred to eBI were given this as the only option and thus did not receive oral advice. This means that half of the patients referred to eBI were also given oral advice using referral to eBI as an adjunct to oral advice. Whether this was due to lack of familiarity with or mistrust of the eBI or simply implies that when referring to eBI, oral advice becomes a part of the referral talk is not known. However, we asked staff to take a few minutes to encourage and motivate patients to log on to the eBI so the latter is highly probable. This is also supported by a somewhat higher log-on rate to the eBI among those who received oral advice and eBI (log-on rate of 22% vs 17%).

Referral to eBI was meant to save time, and on average, the staff spent 12% less time (7.25 min compared with 8.28 min on average) when referring to eBI than when giving oral advice. The reason why more time was not saved could be that staff first had to screen the patients with the pen-and-paper AUDIT-C questionnaire (or in Catalonia, using the electronic record) and then, based on the results, had to explain to the patients why they were recommending that the patient log on to the eBI intervention.

### Differences in the level of implementation in the participating jurisdictions

Differences in the level of implementation among the participating jurisdictions were seen with regard to the proportion of AUDIT-C-positive patients referred to eBI; Poland referred most of their patients (80%) in contrast with the Netherlands where only 10% were referred.

Assuming that a 0 log-on rate reflects the ability to motivate the patients to use the eBI, it seems that patients in Catalonia were less compelled to use the eBI because only 1 of the 12 PHCUs reported a log-on rate >0. We did not study the reason for this, but it could be due to a different culture among patients in Catalonia towards internet use and trust in websites. In the Netherlands, only 5 of the 12 PHCUs reported a log-on rate >0 and 2 PHCUs did not refer a single patient. However, in Poland, 9 of 12 PHCUs reported a log-on rate, 10 of 12 in England and all 12 PHCUs in Sweden (table 1). This might reflect a different maturity in different jurisdictions with regard to eHealth solutions for promoting healthy lifestyle. In Catalonia, this was the first attempt to introduce an eBI. This difference can be expected to fade out in the future but is a real challenge for implementation of eBI in primary care at the present time.

Not much progress has been reported on engagement by healthcare staff in alcohol interventions during the last decade.^19^ ^20^ Thus, there is a need for more translational research that identifies innovative means of embedding alcohol preventive measures into daily practice.^20^ More alcohol eBI implementation studies should therefore be performed to get a more profound understanding of attitudes and practices among providers concerning trust in eBI and how best to support the implementation, including the importance of facilitated access.^21^ ^22^ If successful implementation of an eBI on alcohol could be achieved, there is no doubt that this would save time and money. Simulation studies on the effects of introducing an eBI on alcohol on a larger scale to a whole nation such as the Netherlands show substantial cost-effectiveness for the healthcare system.^26^ But there are still questions to be answered such as whether eBI should be offered after face-to-face screening or whether it is possible to set up a system whereby the patients are referred directly to eBI without face-to-face screening without losing compliance, that is, ensuring that patients do log on to the website.^27^ ^28^

### Strength and limitations

To our knowledge, this is one of the first studies on implementing referral to an alcohol eBI by healthcare staff in primary healthcare. The strength of this study is the participation from five jurisdictions, enabling us to study the variability of referrals to eBI. In addition, the high number of participating providers and PHCUs is seen as a strength. Limitations include the failure of some jurisdictions to implement referral to the eBI as intended, as well as the lack of access and trust in internet-based health promotion among patients (that might be due to the age of the population screened in some jurisdictions). However, the main ODHIN study was not designed to study the reasons for failure in implementing referral to eBI, but a forthcoming qualitative study with participants from the ODHIN project will enable us to answer some of the questions raised by the present study.

## Conclusions

The study shows that it was difficult to get staff engaged in referring to the eBI, a fact that is reflected in the low proportion of staff familiarising themselves with the eBI intervention. The referred patients showed a low level of engagement in logging on to the eBI intervention, although there was a high degree of variation in engagement by patients across and within jurisdictions, perhaps as a consequence of the low level of engagement from the staff, the age of the patients and access to the internet.

What can be learnt from this implementation study? Referral to eBI takes nearly as much time as brief oral advice; less than one-fifth of referred patients actually log on the website; all staff are not ready to refer to eBI and might require more introduction and training before they are comfortable with referring to eBI. Finally, a follow-up routine would also reduce the risk of no advice being delivered.
